# Epidemiological surveillance and phylogenetic diversity of *Orthohantavirus hantanense* using high-fidelity nanopore sequencing, Republic of Korea

**DOI:** 10.1371/journal.pntd.0012859

**Published:** 2025-02-07

**Authors:** Kyungmin Park, Jongwoo Kim, Juyoung Noh, Seong-Gyu Kim, Hee-Kyung Cho, Kijin Kim, Ye-rin Seo, Taehun Lim, Seonghyeon Lee, Jaeyeon Lee, Seung In Lim, Young Hoon Joo, Buddle Lee, Seok Hyeon Yun, Changbo Park, Won-Keun Kim, Jin-Won Song

**Affiliations:** 1 Department of Microbiology, Korea University College of Medicine, Seoul, Republic of Korea; 2 Institute for Viral Diseases, Korea University College of Medicine, Seoul, Republic of Korea; 3 Department of Biomedical Sciences, Graduate Program, Korea University College of Medicine, Seoul, Republic of Korea; 4 Faculty of Health Sciences, Centre for Infectious Disease Genomics and One Health, Simon Fraser University, Burnaby, Canada; 5 Department of Molecular Biology and Biochemistry, Simon Fraser University, Burnaby, Canada; 6 Department of Microbiology, College of Medicine, Hallym University, Chuncheon, Republic of Korea; 7 The Fifth Preventive Medicine Unit of Republic of Korea Army, Pocheon, Republic of Korea; 8 The First Preventive Medicine Unit of Republic of Korea Army, Goyang, Republic of Korea; 9 The Third Preventive Medicine Unit of Republic of Korea Army, Inje, Republic of Korea; 10 The Second Preventive Medicine Unit of Republic of Korea Army, Chuncheon, Republic of Korea; 11 Republic of Korea Army Headquarters, Daejeon, Republic of Korea; 12 Institute of Medical Research, College of Medicine, Hallym University, Chuncheon, Republic of Korea; Chengde Medical University, CHINA

## Abstract

**Background:**

*Orthohantavirus hantanense* (HTNV) poses a substantial global public health threat due to its role in causing hemorrhagic fever with renal syndrome (HFRS). HTNV outbreaks are particularly prevalent in the Gyeonggi and Gangwon Provinces of the Republic of Korea (ROK). This study aimed to evaluate the application of advanced nanopore sequencing and bioinformatics to generate complete genome sequences of HTNV, with the objective of accurately identifying infection sources and analyzing their genetic diversity.

**Methodology/Principal findings:**

In 2022 and 2023, we collected 579 small mammals from 11 distinct locations across Gyeonggi and Gangwon Provinces, as well as in Gwangju Metropolitan City, ROK. Among these, 498 *Apodemus agrarius* specimens were subjected to an epidemiological survey to investigate HTNV infections. The serological and molecular positivity of HTNV were found to be 65/498 (13.1%) and 17/65 (26.2%), respectively. Furthermore, 15 whole-genome sequences of HTNV were obtained from rodents in Gyeonggi and Gangwon Provinces. We developed a novel amplicon-based nanopore sequencing approach to acquire high-fidelity and precise genomic sequences of HTNV. Genome exchange analysis revealed three reassortant candidates, including heterogeneous L segments, from Paju-si and Yeoncheon-gun in Gyeonggi Province.

**Conclusion/Significance:**

Our findings enhance the resolution of the spatiotemporal genomic surveillance of HTNV by consistently providing new viral sequences and epidemiological data from HFRS-endemic regions in the ROK. This report signifies a notable advancement in nanopore sequencing techniques and bioinformatics, offering a robust platform for genome-based diagnostics and sophisticated phylogenetic analyses of orthohantaviruses, which are essential for public health strategies aimed at controlling HFRS.

## Author summary

Whole-genome sequencing is pivotal in unraveling the complexities of viral evolution, genomic epidemiology, and the transmission dynamics of emerging infectious agents. Oxford Nanopore Technologies (ONT) offers a portable and cost-effective platform that has become invaluable for virological studies. *Orthohantavirus hantanense* (HTNV), the causative agent of hemorrhagic fever with renal syndrome (HFRS), represents a significant global public health concern. In the Republic of Korea (ROK), HTNV outbreaks are especially prevalent in the Gyeonggi and Gangwon Provinces, where the striped field mouse (*Apodemus agrarius*) serves as the primary reservoir host. To obtain high-fidelity genomic data, an advanced amplicon-based nanopore sequencing method was developed, utilizing the latest R10 chemistry and KU-ONT-HTNV-consensus modules. This approach led to the generation of 15 complete HTNV genomes from rodents. These advancements have substantially improved the spatiotemporal genomic surveillance of HTNV, facilitating high-resolution phylogeographic and epidemiological analyses, and deepening our understanding of viral phylodynamics. This report provides valuable insights into nanopore-based diagnostics, genomic surveillance, and the evolutionary history of orthohantaviruses, all of which are essential for formulating effective control strategies against HFRS in endemic regions of the ROK.

## Introduction

Orthohantaviruses, members of the *Bunyavirales* order and the *Hantaviridae* family, are enveloped, single-stranded, negative-sense RNA viruses [1]. Their genomes are segmented into three RNA segments: small (S), medium (M), and large (L), which encode a nucleocapsid protein, two surface glycoproteins (Gn and Gc), and an RNA-dependent RNA polymerase, respectively. Some orthohantaviruses are zoonotic pathogens that cause hemorrhagic fever with renal syndrome (HFRS) in Eurasia and hantavirus cardiopulmonary syndrome in the Americas [2]. In East Asia alone, approximately 150,000 cases of HFRS are reported annually, with mortality rates ranging from <1 to 15% [3]. HFRS is mainly attributed to infections by *Orthohantavirus hantanense* (HTNV), *Orthohantavirus seoulense* (SEOV), *Orthohantavirus dobravaense* (DOBV), and *Orthohantavirus puumalaense* (PUUV) [4]. The natural reservoirs of hantaviruses include various species of rodents, shrews, moles, and bats, reflecting a long-standing evolutionary co-divergence with their primary hosts [5–7]. Human transmission of orthohantaviruses typically occurs through inhalation of aerosolized viral particles from the saliva, urine, and feces of infected rodents [8].

Approximately 400 cases of HFRS are reported annually in the Republic of Korea (ROK), with mortality rates ranging from 1% to 4%. Hantavirus outbreaks are particularly common in the northern areas of Gyeonggi and Gangwon Provinces, affecting both military personnel and civilian populations [9,10]. Recently, a novel phylogenetic lineage of HTNV, associated with HFRS, was discovered in patients and rodents in southern regions of the ROK, including Gwangju Metropolitan City, Boseong-gun, and Jeju Island [11,12]. Previous studies have emphasized the importance of genome-based analyses of HTNV in elucidating the phylogenetic and epidemiological relationships between HFRS patients and their rodent hosts, which is critical for identifying potential sites of viral exposure [13]. For instance, in 2005, clinical sequencing of HTNV from four United States (US) Army soldiers who contracted HFRS while serving in the US Forces Korea (USFK) revealed a phylogeographic correlation between the viral sequences and those found in rodents trapped at military training locations where the soldiers had been active [14]. Further genomic epidemiological studies conducted between 2013 and 2015, which analyzed nearly complete HTNV genome sequences from soldiers of both the ROK and USFK, reinforced these connections [15]. The expansion of viral genome databases has been instrumental in developing public health strategies for HFRS by clarifying the epidemiological links between patients and the sources of infection [4].

Next-generation sequencing (NGS) technologies play crucial roles in advancing various areas of virology, including whole-genome sequencing, metagenomics, tracking transmission dynamics, exploring the genome epidemiology of infectious agents, and aiding in vaccine development [16]. The ability to sequence the complete genomes of emerging viruses is especially important for clinical diagnosis, virulence evaluation, and source identification during disease outbreaks [17,18]. However, a significant challenge in obtaining full-length genomic sequences is the extremely low concentration of viral genomes in clinical or environmental samples. To address this, several NGS approaches have been developed, such as sequence-independent single-primer amplification, targeted enrichment of viral genomes using specific oligonucleotide probes, and amplicon-based NGS [15,19,20]. These approaches improve the detection and sequencing of viral genomes, allowing researchers to generate comprehensive and accurate genomic data, even when viral genome copies are scarce in samples.

The MinION sequencer (Oxford Nanopore Technologies [ONT], London, UK) is a portable device that utilizes long-read sequencing technology and offers a compact and cost-effective alternative to traditional NGS systems [21]. This nanopore-based platform is particularly valuable for point-of-care testing of various viral infections, including Zika, Chikungunya, Ebola, hepatitis C, and hepatitis A viruses, enabling real-time sequencing of clinical samples [22–25]. In hantavirus research, the performance and effectiveness of nanopore sequencing for obtaining full-length genomic sequences have been evaluated using two New World hantavirus species: *Orthohantavirus sinnombreense* (SNV) and *Orthohantavirus prospectense* [26]. Furthermore, amplicon-based MinION sequencing has been investigated as a diagnostic tool to quickly produce nearly complete genomic sequences of HTNV and SEOV from patients with HFRS and their primary reservoir hosts [11,27,28]. This approach substantially reduces the sequencing time compared with the Illumina platform, providing a faster alternative for genomic analysis. However, these technologies face challenges related to reliability owing to high error rates in early nanopore systems, which induce mechanical insertion and deletion (indel) errors, particularly in homopolymer regions, with earlier R9 chemistry [29–32].

In this study, an epidemiological surveillance was conducted on small mammals to determine the serological and molecular positivity of HTNV in endemic regions of HFRS in the ROK between 2022 and 2023. We evaluated the potential of advanced nanopore sequencing and bioinformatic approaches to obtain high-ﬁdelity complete genome sequences of HTNV, with the goal of accurately identifying infectious sources and assessing the genetic diversity of the variants. This report provides valuable insights into the nanopore-based diagnostics, genome epidemiology, and evolutionary history of orthohantaviruses, which are essential for developing effective strategies to mitigate HFRS outbreaks in the ROK.

## Methods

### Ethics statement

The handling of small mammals followed the ethical guidelines established by the Korea University Institutional Animal Care and Use Committee (#2022‐34). Autopsies and laboratory experiments were conducted in an animal biosafety level 3 (ABSL3) facility at the Korea University College of Medicine, Seoul, ROK.

### Animal trapping and sample collection

Rodents and shrews were collected from 11 areas, including Pocheon-si, Paju-si, Yeoncheon-gun, and Yangju-si in Gyeonggi Province; Chuncheon-si, Cheorwon-gun, Hongcheon-gun, Inje-gun, Hwacheon-gun, and Yanggu-gun in Gangwon Province; and Gwangju Metropolitan City, ROK. Animal trapping was performed using Sherman live traps (8 × 9 × 23 cm; H. B. Sherman) in 2022 and 2023. The capture and transportation procedures have been previously detailed [33]. Multiple tissues and sera were aseptically collected in a biosafety cabinet in ABSL3 laboratory and then stored at −80°C until use.

### Mitochondrial DNA analysis

Total DNA was extracted from liver tissues homogenized using TRI Reagent Solution (Ambion, Austin, TX, USA) following the manufacturer’s protocol. To verify the taxonomic classification of the small mammals, a polymerase chain reaction (PCR) targeting the mitochondrial DNA cytochrome *b* (*CYTB*) gene was performed. The PCR utilized universal primers and cycling conditions as previously described [34].

### Indirect immunofluorescence antibody test

Sera and heart fluids were subjected to centrifugation at 4°C for 5 min to achieve separation. The sera (1:32) and heart fluids were diluted (1:2) in phosphate-buffered saline (PBS) before being applied to acetone-fixed Vero E6 cells infected with HTNV. The plates were incubated at 37°C for 30 min. After incubation, the wells were thoroughly washed with PBS followed by distilled water. The cells were then treated with fluorescein isothiocyanate-conjugated goat antibodies against mouse immunoglobulin G (IgG; for rodents) or mouse/rat IgG (for shrews) (MP Bio, CA, USA). Slides were incubated at 37°C for 30 min and subsequently washed again. Hantavirus-specific fluorescence was observed using a fluorescence microscope (Axio Scope, Zeiss, Berlin, Germany). Antibody titers were determined as the highest dilution factor of the sample that displayed weak, but specific fluorescence, based on a two-fold serial dilution.

### RT-PCR

Total RNA was extracted from the homogenized lung tissues of seropositive rodents using TRI Reagent Solution (Ambion), following the manufacturer’s protocol. cDNA synthesis was performed using 1 μg of total RNA with the High Capacity RNA-to-cDNA Kit (Applied Biosystems, Foster City, CA, USA), utilizing a random hexamer and OSM55 (5′-TAG TAG TAG ACT CC-3′). The first and second rounds of PCR were conducted in 25-μL reaction volumes containing 0.625 U of Ex Taq DNA polymerase (TaKaRa BIO, Tokyo, Japan), 2.5 μL of 10X Ex Taq buffer, 2 μL of a dNTP mixture (2 mM each), 10 pmol of each primer (final concentration: 0.4 μM), and 1.5 μL of the cDNA template. The oligonucleotide primer sequences used have been previously described [35]. The cycling conditions included an initial denaturation at 94°C for 5 min, followed by a first stage of six cycles comprising denaturation at 94°C for 30 s, annealing at 37°C for 40 s, and elongation at 72°C for 1 min. This was followed by a second stage of 32 cycles with denaturation at 94°C for 30 s, annealing at 42°C for 40 s, and elongation at 72°C for 1 min, concluding with a final elongation step at 72°C for 5 min. The PCR was conducted using a ProFlex PCR System (Life Technologies, Carlsbad, CA, USA). PCR products were purified using the MinElute PCR Purification Kit (Qiagen, Hilden, Germany) and amplicon sequencing was performed using the BigDye Terminator v3.1 Cycle Sequencing Kit (Applied Biosystems) on an automated sequencer (ABI 3730XL DNA Analyzer; Applied Biosystems).

### Quantitative PCR (qPCR)

qPCR was carried out in a reaction volume of 10 μL, containing 1 μL of cDNA generated from total RNA (as described in the RT-PCR section), using the SYBR Green PCR Master Mix (Applied Biosystems) on a QuantStudio 5 Flex real-time PCR system (Applied Biosystems). The thermal cycling protocol commenced with an initial denaturation at 95°C for 10 min, followed by 45 cycles of amplification, each consisting of 95°C for 15 s and 60°C for 1 min. The primers used have been described previously [36]. Viral copy numbers were determined by generating a linear regression curve from recombinant plasmid DNA containing the S segment of HTNV, as described previously [20].

### Amplicon-based nanopore sequencing

Total RNA was extracted from the lung tissues of rodents positive for HTNV RNA using TRI Reagent Solution (Ambion), followed by treatment with ezDNase (Invitrogen, Carlsbad, CA, USA) according to the manufacturer’s instructions. After RNA extraction, a one-step RT-PCR was conducted using the SuperScript IV one-step RT-PCR system (Invitrogen), following in-house standard protocols for amplifying HTNV genomes with universal primers [37]. The amplicons were then pooled and prepared using the Native Barcoding Kit 24 V14 (SQKNBD114.24; ONT) according to the manufacturer’s guidelines. End-prepared libraries were purified using AMPure XP beads (Beckman Coulter, CA, USA). Following purification, the barcoded libraries were pooled, ligated to sequencing adapters, and subsequently sequenced on an MK1C device (ONT) using a FLO-MIN114 (R10.4.1) flow cell. Sequencing was performed for 30 min per sample.

### NGS data analysis

Raw data were processed using Guppy (v.6.1.5) embedded in the MK1C system (ONT), followed by real-time base-calling in the high-accuracy mode, removal of adaptor sequences, and filtering of reads between 20 bp and 2 kb, ensuring a minimum Q-score greater than nine for subsequent analyses. The filtered reads were merged and converted into a single FASTQ file using Porechop (v.9.0). Consensus sequences were generated using the KU-ONT-HTNV-consensus module (available at https://github.com/KijinKims/KU-ONT-HTNV-consensus), which enabled the alignment of reads to each segment of the HTNV 76-118 reference genome. Genomic variants were detected using Medaka (https://github.com/nanoporetech/medaka) and filtered according to variant quality and sequencing depth using BCFtools [38]. To correct mechanical indel errors at the homopolymer sites, particularly those affecting minor variant reads within the alignment, genome polishing was performed using a customized in-house Python script. The final consensus sequences were derived from the identified variants in relation to the reference genome, with positions lacking sufficient coverage depth (below the minimum threshold of 5) excluded and marked as “N” using BED tools [39].

### Phylogenetic analysis

The genomic sequences of the HTNV S, M, and L segments were aligned using the ClustalW method in MegAlign software (Lasergene v.5; DNASTAR, Madison, WI, USA). Phylogenetic trees were subsequently reconstructed using the maximum likelihood approach in MEGA 7 [40], with the optimal substitution models selected for each segment: T92+G for the S segment, T92+G+I for the M segment, and TN93+G+I for the L segment. The robustness of the resulting topologies was determined using bootstrap analysis with 1,000 iterations. For high-resolution phylogenetic analysis, the newly acquired viral sequences were analyzed alongside full-length genome sequences from Gyeonggi and Gangwon Provinces in the ROK, which are geographically close to the study area. These sequences were retrieved from NCBI. Additionally, HV004 from China and HTNV Ac20-5 from Jeju Island in the ROK were included as outgroups. The reference sequences used in this study are listed in S1 Table.

### Genetic reassortment analysis

Genetic reassortment events were evaluated using the graph incompatibility-based reassortment finder (GiRaF) [41]. Alignments of HTNV tripartite genomes were used as inputs for Bayesian inference analysis [42]. In total, 1,000 unrooted candidate phylogenies were generated under the GTR+G+I substitution model, incorporating a burn-in of 25% (50,000 iterations) and sampling every 200 iterations. These trees were employed to account for the evolutionary uncertainty across each segment, following the default settings in GiRaF. The default confidence threshold was maintained to 0.7. This procedure was repeated 10 times, producing 10 independent MrBayes tree files for each segment.

## Results

### Trapping for small mammals in the ROK in 2022–2023

In 2022 and 2023, we collected 579 small mammals from 11 distinct areas: Pocheon-gun, Paju-si, Yeoncheon-gun, and Yangju-si in Gyeonggi Province; Chuncheon-si, Cheorwon-gun, Hongcheon-gun, Inje-gun, Hwacheon-gun, and Yanggu-gun in Gangwon Province; and Gwangju Metropolitan City, ROK (S1 Fig). The captured rodents and shrews consisted of 498 *Apodemus agrarius*, four *A. peninsulae*, 42 *Crocidura lasiura*, seven *C. shantungensis*, one *Sorex mirabilis*, 17 *Myodes regulus*, five *Tscherskia triton*, four *Micromys minutus*, and one *Tamias sibiricus* (S2 Fig). The species identity of *A. agrarius* was confirmed through phylogenetic analysis of their mitochondrial DNA *CYTB* gene (S3 Fig).

### Epidemiological survey of HTNV

In total, 65/498 (13.1%) *A. agrarius* tested positive for anti-HTNV IgG, of which 7/36 (19.4%), 18/95 (18.9%), 12/68 (17.6%), 2/16 (12.5%), 15/124 (12.1%), 3/38 (7.9%), 3/40 (7.5%), 2/39 (5.1%), and 3/20 (15%) were captured in Pocheon-gun, Paju-si, Yeoncheon-gun in Gyeonggi Province; Chuncheon-si, Cheorwon-gun, Hongcheon-gun, Inje-gun, and Hwacheon-gun in Gangwon Province; and Gwangju Metropolitan City, respectively (Table 1). No seropositive rodents were detected in Yangju-si, Gyeonggi Province, or Yanggu-gun, Gangwon Province. In total, of 17/65 (26.2%) *A. agrarius* were found to carry HTNV RNA, including 4/18 (22.2%) from Paju-si, 5/12 (41.7%) from Yeoncheon-gun, 6/15 (40%) from Cheorwon-gun, 1/3 (33.3%) from Inje-gun, and 1/2 (50%) from Hwacheon-gun. No hantaviral RNA-positive rodents were identified in Pocheon-si, Chuncheon-si, Hongcheon-gun, or Gwangju Metropolitan City using RT‐PCR. The epidemiological characteristics of HTNV-infected *A. agrarius* are shown in S2 Table.

**Table 1:** Serological and molecular positivity of *Orthohantavirus hantanense* (HTNV) in *Apodemus agrarius* collected in the Republic of Korea, 2022–2023.

Collection site		Seropositive rate for anti- HTNV IgG antibody (%)	HTNV RNA positivity (%)^a^
GyeonggiProvince	Pocheon-gun	7/36 (19.4)	0/7
	Paju-si	18/95 (18.9)	4/18 (22.2)
	Yeoncheon-gun	12/68 (17.6)	5/12 (41.7)
	Yangju-si	0/6	n.d
**Subtotal**		**37/205 (18.0)**	**9/37 (24.3)**
Gangwon Province	Chuncheon-si	2/16 (12.5)	0/2
	Cheorwon-gun	15/124 (12.1)	6/15 (40)
	Hongcheon-gun	3/38 (7.9)	0/3
	Inje-gun	3/40 (7.5)	1/3 (33.3)
	Hwacheon-gun	2/39 (5.1)	1/2 (50)
	Yanggu-gun	0/16	n.d
**Subtotal**		**25/273 (9.2)**	**8/25 (32)**
Gwangju Metropolitan City		3/20 (15)	0/3
**Subtotal**		**3/20 (15)**	**0/3**
**Total**		**65/498 (13.1)**	**17/65 (26.2)**

^a^The positivity of HTNV RNA was determined using nested reverse transcription-polymerase chain reaction.

IgG, immunoglobulin G; n.d, not determined

### Coverage rate and average depth of nanopore sequencing for HTNV by viral copy numbers

HTNV RNA copy numbers were determined from the cycle threshold values in the lung tissues 17 *A. agrarius* (Table 2). Using one-step RT-PCR-based nanopore sequencing, nearly all full-length genomic sequences of HTNV were recovered from the lung tissues of HTNV‐positive *A. agrarius* collected in the ROK between 2022 and 2023 (S4 Fig). Aa22-65 exhibited the highest viral copy numbers, with genome coverage of 98.3% for the S segment, 99.2% for the M segment, and 95.6% for the L segment. Among seven rodents (Aa23-132, Aa22-84, Aa23-34, Aa23-130, Aa22-95, Aa23-35, and Aa23-117) with viral RNA copies ranging from 10^6^ to 10^7^ copies/μL, genome coverage ranged from 98.3% for the S segment, 99.1% to 99.2% for the M segment, and 77.9% to 99.6% for the L segment. Aa23-118, with viral RNA copies ranging from 10^5^ to 10^6^ copies/μL, demonstrated genome coverage of 98.3% for the S segment, 99.1% for the M segment, and 99.3% for the L segment. Aa22-159 and Aa22-82 had viral RNA copies ranging from 10^4^ to 10^5^ copies/μL, resulting in genome coverage of 98.3% for the S segment, 99.2% for the M segment, and 99.5% to 99.6% for the L segment. Three individuals (Aa23-170, Aa22-127, and Aa23-115) with viral RNA copies ranging from 10^3^ to 10^4^ copies/μL achieved genome coverage of 98.3% for the S segment, 99.2% for the M segment, and 92.2% to 99.6% for the L segment. With viral RNA copies ranging from 10^2^ to 10^3^ copies/μL, Aa22-184 revealed 98.3% for the S segment and 99.2% genome coverage for the L and M segments. Aa23-89, with viral RNA copies ranging from 10 to 10^2^ copies/μL, displayed genome coverage of 98.3% for the S segment, 99.2% for the M segment, and 72.5% for the L segment. The average number of viral reads mapped to the reference genomic sequence (HTNV 76-118 strain) was 11,755 (17.6%) for the S segment, 19,321 (29.0%) for the M segment, and 21,354 (32.0%) for the L segment, with an average depth of coverage of 5,587 for the S segment, 3,669 for the M segment, and 2,443 for the L segment (S3 Table).

**Table 2:** Genome coverage rates of multiplex polymerase chain reaction‐based nanopore sequencing relative to viral RNA copy number of *Orthohantavirus hantanense* (HTNV).

Viral RNA copy number (copies/uL)	Sample	Collection site	Origin	Ct value	HTNV genomes, % coverage (minimum depth,>5X)		
					S segment	M segment	L segment
10^7^ to 10^8^	Aa22-65	Inje-gun	Lung	14.8	98.3	99.2	95.6
10^6^ to 10^7^	Aa23-132	Cheorwon-gun	Lung	16.5	98.3	99.2	99.6
	Aa22-84	Paju-si	Lung	16.7	98.3	99.2	99.3
	Aa23-34	Yeoncheon-gun	Lung	16.7	98.3	99.2	77.9
	Aa23-130	Cheorwon-gun	Lung	16.9	98.3	99.2	99.6
	Aa22-95	Paju-si	Lung	17.4	98.3	99.2	99.6
	Aa23-35	Yeoncheon-gun	Lung	17.4	98.3	99.1	84.7
	Aa23-117	Cheorwon-gun	Lung	19.1	98.3	99.2	94.8
10^5^ to 10^6^	Aa23-118	Cheorwon-gun	Lung	19.6	98.3	99.1	93.3
10^4^ to 10^5^	Aa22-159	Cheorwon-gun	Lung	22.6	98.3	99.2	99.5
	Aa22-82	Yeoncheon-gun	Lung	24.9	98.3	99.2	99.6
10³ to 10^4^	Aa23-170	Paju-si	Lung	25.1	98.3	99.2	99.6
	Aa22-127	Hwacheon-gun	Lung	26.1	98.3	99.2	99.4
	Aa23-115	Cheorwon-gun	Lung	26.1	98.3	99.2	92.2
10² to 10³	Aa23-174	Paju-si	Lung	29.3	98.3	99.2	75.1
	Aa22-184	Yeoncheon-gun	Lung	29.6	98.3	99.2	99.2
10 to 10²	Aa23-89	Yeoncheon-gun	Lung	31.7	98.3	99.2	72.5

Ct, cycle threshold; Aa, *Apodemus agrarius*

### Whole-genome sequencing of HTNV

**Fig 1:**
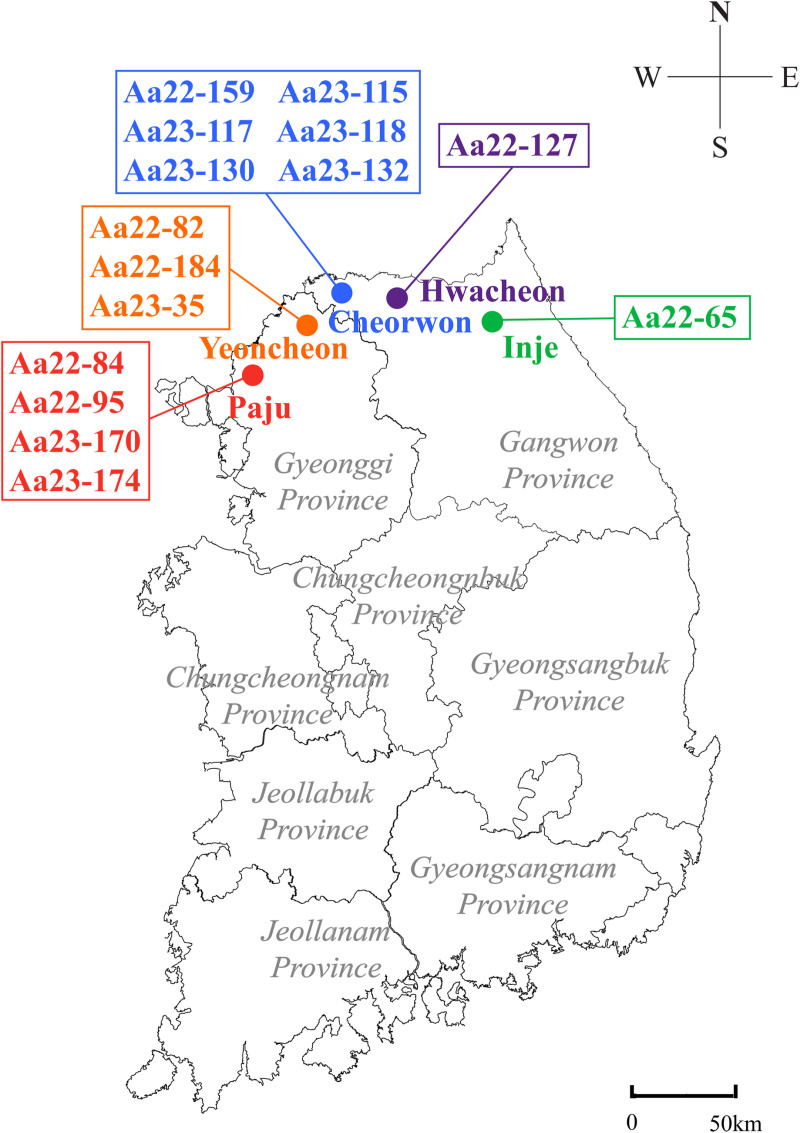
Geographical locations where complete-genomic sequences of *Orthohantavirus hantanense* (HTNV) were acquired from rodents captured in the Republic of Korea (ROK), in 2022–2023. A total of 15 entire-genomic sequences of HTNV were obtained from Striped filed mice (*Apodemus agrarius*) collected from Gyeonggi and Gangwon Provinces, ROK, between 2022 and 2023. The colored circles represent the HTNV RNA positive sites: red, Paju-si (for Aa22-84, Aa22-95, Aa22-170, and Aa23-174); orange, Yeoncheon-gun (for Aa22-82, Aa22-184, and Aa23-35); blue, Cheorwon-gun (for Aa22-159, Aa23-115, Aa23-117, Aa23-118, Aa23-130, and Aa23-132); green, Inje-gun (for Aa22-65); purple, Hwacheon-gun (for Aa22-127), respectively. The initial map was created using a Quantum Geographical Information System 3.10 for Mac and modified using Adobe Illustrator CC 2019. The base layer of the map was sourced from https://www.naturalearthdata.com/ and is freely available for use in any project without the need for permission.

Fifteen complete genome sequences of HTNV were retrieved from rodents captured in five distinct regions: Paju-si and Yeoncheon-gun in Gyeonggi Province; Cheorwon-gun, Inje-gun, and Hwacheon-gun in Gangwon Province, ROK, in 2022–2023 (Fig 1). The 3′- and 5′-termini genome sequences were empirically obtained due to the conserved areas within the *Hantaviridae* family [43].

### High-resolution phylogenetic analysis of HTNV

**Fig 2:**
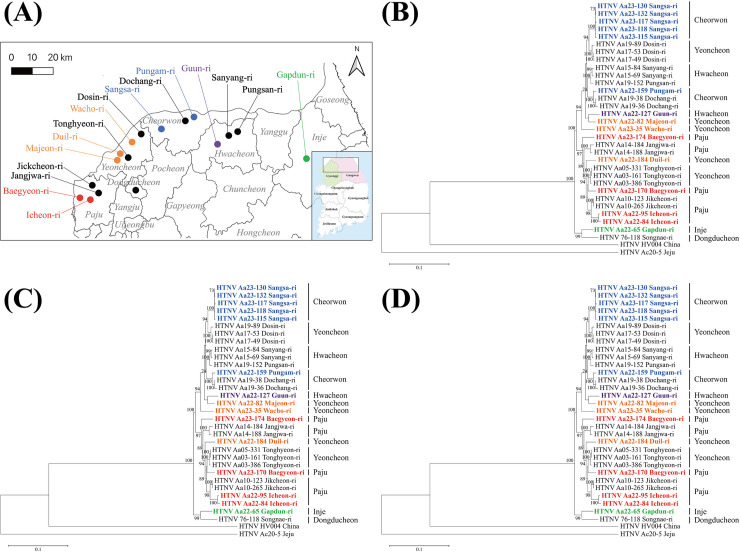
High-resolution phylogenetic analysis of the S, M, and L segments of *Orthohantavirus hantanense* (HTNV) from rodents collected in the Republic of Korea (ROK), 2022–2023. (A) The geographical locations where the newly acquired HTNV genomes were collected are marked with colored circles on the map, representing different regions. The initial map was created using a Quantum Geographical Information System 3.10 for Mac and modified using Adobe Illustrator CC 2019. The base layer of the map was sourced from https://www.naturalearthdata.com/ and is freely available for use in any project without the need for permission. The phylogenetic trees were constructed using the maximum likelihood method in MEGA 7, employing the best-fit evolutionary model based on the (B) HTNV S segment (positions 1–1,696 nt), (C) M segment (positions 1–3,616 nt), and (D) L segment (positions 1–6,530 nt), respectively. In this figure, the genomic sequences of HTNV are displayed, with newly obtained sequences highlighted in distinct colors: red for Paju (Baegyeon-ri and Icheon-ri), orange for Yeoncheon (Majeon-ri, Duil-ri, and Wacho-ri), blue for Cheorwon (Pungam-ri and Sangsa-ri), green for Inje (Gapdun-ri), and purple for Hwacheon (Guun-ri). The branch lengths in the phylogenetic tree correspond to the number of nucleotide substitutions, while the vertical distances are adjusted to enhance clarity. Bootstrap probabilities, calculated from 1,000 iterations, are indicated at each node. The accession numbers of HTNV used in this study are shown in S1 Table.

A high-resolution geographic map illustrating the collection sites of the newly acquired HTNV genomes in this study is shown in **Fig 2A**. Phylogenetic analysis was conducted using the full-length genomic sequences of HTNV S segments acquired from five regions: Paju-si (Baegyeon-ri and Icheon-ri), Yeoncheon-gun (Majeon-ri, Duil-ri, and Wacho-ri), Cheorwon-gun (Pungam-ri and Sangsa-ri), Hwacheon-gun (Guun-ri), and Inje-gun (Gapdun-ri) (Fig 2B). Phylogenetically, the HTNV S segment sequences from samples Aa23-170 and Aa23-174 from Paju-si (Baegyeon-ri) were grouped with Aa22-184 from Yeoncheon-gun (Duil-ri), whereas Aa22-84 and Aa22-95 from Paju-si (Icheon-ri) formed a distinct genetic lineage that was closely related to variants from Paju-si (Jikcheon-ri). The HTNV S segment from sample Aa22-82, collected in Yeoncheon-gun (Majeon-ri), were clustered genetically with strains from Hwacheon-gun (Sanyang-ri, Pungsan-ri, and Guun-ri). The S segment of sample Aa23-35 from Yeoncheon-gun (Wacho-ri) shared a common ancestor with those collected from Yeoncheon-gun (Dosin-ri). The S segment sequences from the five strains in Cheorwon-gun (Sangsa-ri) exhibited an independent lineage that was distinct from the others in Yeoncheon-gun (Majeon-ri) and Hwacheon-gun (Sanyang-ri, Pungsan-ri, and Guun-ri). The HTNV S segment from sample Aa22-159 in Cheorwon-gun (Pungam-ri) clustered with Aa19-38 from Cheorwon-gun (Dochang-ri), whereas the S segment from Aa22-127 in Hwacheon-gun (Guun-ri) shared a common ancestor with variants from Hwacheon-gun (Sanyang-ri and Pungsan-ri). Additionally, the HTNV S segment from sample Aa22-65 collected in Inje-gun (Gapdun-ri) formed an independent clade distinct from those collected in Paju-si, Yeoncheon-gun, Cheorwon-gun, and Hwacheon-gun, except for the HTNV 76-118 strain.

A phylogenetic tree was constructed using the full-length genomic sequences of the HTNV M segment obtained from five distinct locations: Paju-si (Baegyeon-ri and Icheon-ri), Yeoncheon-gun (Majeon-ri, Duil-ri, and Wacho-ri), Cheorwon-gun (Pungam-ri and Sangsa-ri), Hwacheon-gun (Guun-ri), and Inje-gun (Gapdun-ri) (Fig 2C). Phylogenetically, the HTNV M segment sequences from Aa23-170 and Aa23-174 in Paju-si (Baegyeon-ri) formed an independent lineage along with other sequences from Paju-si (Jangjwa-ri, Jikcheon-ri, and Icheon-ri), Yeoncheon-gun (Wacho-ri, Majeon-ri, Dosin-ri, and Tonghyeon-ri), Cheorwon-gun (Pungam-ri, Sangsa-ri, and Dochang-ri), and Hwacheon-gun (Sanyang-ri, Pungsan-ri, and Guun-ri). Conversely, sequences from Aa22-84 and Aa22-95 (Icheon-ri) clustered with strains from Paju-si (Jikcheon-ri). The HTNV M segment from Aa22-82, collected from Yeoncheon-gun (Majeon-ri), was genetically associated with the strains from Yeoncheon-gun (Wacho-ri and Dosin-ri), Cheorwon-gun (Sangsa-ri, Pungam-ri, and Dochang-ri), and Hwacheon-gun (Sanyang-ri, Pungsan-ri, and Guun-ri). Similarly, the genomic sequence of the HTNV M segment from Aa22-184 in Yeoncheon-gun (Duil-ri) formed an independent lineage grouped with sequences from Paju-si (Baegyeon-ri, Jangjwa-ri, Jikcheon-ri, and Icheon-ri), Yeoncheon-gun (Wacho-ri, Dosin-ri, and Tonghyeon-ri), Cheorwon-gun (Sangsa-ri, Pungam-ri, and Dochang-ri), and Hwacheon-gun (Sanyang-ri, Pungsan-ri, and Guun-ri). The HTNV M segment from Aa23-35, collected from Yeoncheon-gun (Wacho-ri), clustered with the strains from Cheorwon-gun (Sangsa-ri), and Yeoncheon-gun (Dosin-ri). The M segment from Aa22-159 in Cheorwon-gun (Pungam-ri) shares a common ancestor with Aa19-38 in Cheorwon-gun (Dochang-ri). The M segment from five variants in Cheorwon-gun (Sangsa-ri) formed a genetic clade closely related to HTNV strains from Yeoncheon-gun (Dosin-ri). Finally, the HTNV M segment from Aa22-127, collected from Hwacheon-gun (Guun-ri), was clustered with the strains from Hwacheon-gun (Sanyang-ri and Pungsan-ri). The HTNV M segment from Aa22-65, collected from Inje-gun (Gapdun-ri), formed a phylogenetic lineage with the HTNV 76-118 strain.

Phylogenies were reconstructed using full-length genomic sequences of the HTNV L segment obtained from several locations: Paju-si (Baegyeon-ri and Icheon-ri), Yeoncheon-gun (Majeon-ri, Duil-ri, and Wacho-ri), Cheorwon-gun (Pungam-ri and Sangsa-ri), Inje-gun (Gapdun-ri), and Hwacheon-gun (Guun-ri) (Fig 2D). Phylogenetically, the HTNV L segment sequence from Aa23-170 from Paju-si (Baegyeon-ri) was clustered with strains from Yeoncheon-gun (Tonghyeon-ri), whereas Aa23-174 from Paju-si (Baegyeon-ri) formed a distinct genetic group within Paju-si (Jangjwa-ri, Jikcheon-ri, and Icheon-ri) and Yeoncheon-gun (Tonghyeon-ri and Duil-ri). The HTNV L segment sequences from Aa22-84 and Aa22-95 collected from Paju-si (Icheon-ri) were genetically linked with those from Paju-si (Jikcheon-ri). The genomic sequence of the HTNV L segment from Aa22-82 in Yeoncheon-gun (Majeon-ri) grouped with the Aa22-127 strain from Hwacheon-gun (Guun-ri), whereas Aa23-35 from Yeoncheon-gun (Wacho-ri) formed an independent lineage alongside sequences from Yeoncheon-gun (Dosin-ri and Majeon-si), Cheorwon-gun (Sangsa-ri, Pungam-ri, and Dochang-ri), and Hwacheon-gun (Sanyang-ri, Pungsan-ri, and Guun-ri). The HTNV L segment from Aa22-159 in Cheorwon-gun (Pungam-ri) shared a common ancestor with strains from Cheorwon-gun (Dochang-ri). Additionally, the L segment from five strains from Cheorwon-gun (Sangsa-ri) displayed a phylogenetic lineage closely related to HTNV variants from Yeoncheon-gun (Dosin-ri). Finally, the HTNV L segment from Aa22-65 in Inje-gun (Gapdun-ri) formed a genetic clade with the HTNV 76-118 strain.

### Genome exchange analysis of HTNV

**Fig 3:**
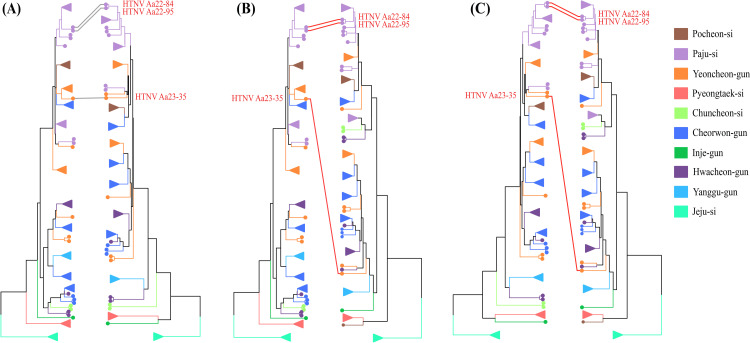
Detection of potential reassortment events among *Orthohantavirus hantanense* (HTNV) genomes obtained in this study. Genetic reassortment was assessed using a graph incompatibility-based reassortment finder, with alignments of whole-genome sequences of HTNV S, M, and L segments serving as the input for Bayesian inference. A total of 1,000 unrooted candidate trees were constructed using the GTR+G+I substitution model, with a burn-in of 25% (50,000 iterations) and sampling every 200 iterations to account for evolutionary uncertainty across each segment. To simplify the comparison between the dendrograms of the phylogenies, the dendextend package in R was used before identifying the incompatible splits among the tripartite genomes of HTNV. The geographical locations where HTNV genomes were sampled are represented by colored circles and collapsed funnel symbols within the figure for clarity and enhanced visualization. Reassortment events are indicated by red auxiliary lines connecting the phylogenetic trees of the (A) S and M, (B) S and L, and (C) M and L segments. Three instances of genetic exchange involving HTNV strains Aa22-84, Aa22-95, and Aa23-35 were predicted across all samples, with confidence values exceeding 0.9 across all samples.

The aligned genomic sequences of HTNV S, M, and L segments were analyzed to infer reassortment events using GiRaF. Genome exchange analysis revealed two reassortments between the S-L and M-L segments in Aa22-84 and Aa22-95 from Paju-si, and Aa23-35 from Yeoncheon-gun, Gyeonggi Province (Fig 3 and Table 3). Confidence levels exceeding 0.9 were observed in all samples.

**Table 3:** Results of genetic reassortment analysis for each segment of *Orthohantavirus hantanense* (HTNV) obtained in this study.

No.	Sample	Collection site (City/Province)	S-M	S-L	M-L	Reassortment segment
1	Aa22-84	Paju-si/Gyeonggi	No	Yes	Yes	L
2	Aa22-95	Paju-si/Gyeonggi	No	Yes	Yes	L
3	Aa23-35	Yeoncheon-gun/Gyeonggi	No	Yes	Yes	L
**Total**			**0**	**3**	**3**	

Genetic reassortment events were evaluated by using a graph incompatibility-based reassortment finder. Alignments of HTNV tripartite genomes were used as inputs for Bayesian inference. A total of 1,000 unrooted candidate trees were generated using the GTR+G+I substitution model with a burn-in rate of 25% (50,000 iterations), and sampling was conducted every 200 iterations. These phylogenies account for evolutionary uncertainty across each segment. This process was repeated 10 times, producing 10 independent MrBayes tree files per segment. Confidence values exceeding 0.9 were observed in all samples. S, small; M, medium; L, large

## Discussion

In this study, 15 whole-genome sequences of HTNV were obtained from rodents captured from five distinct locations within the Gyeonggi and Gangwon provinces: Paju (Baegyeon-ri and Icheon-ri), Yeoncheon (Majeon-ri, Duil-ri, and Wacho-ri), Cheorwon (Pungam-ri and Sangsa-ri), Inje (Gapdun-ri), and Hwacheon (Guun-ri). These sequences have substantially enhanced our phylogenetic understanding of orthohantaviruses in regions of the ROK where HFRS is endemic. Notably, even within a single geographic location, different strains exhibited various phylogenetic branching patterns, highlighting the genetic diversity of orthohantaviruses in the ROK. This study also underscores the importance of ongoing surveillance to monitor and mitigate the risk of HFRS outbreaks, particularly in newly identified endemic areas that have not been previously reported. The improved phylogenetic resolution provides valuable insights for shaping public health strategies aimed at HFRS prevention and strengthening disease management in emerging risk regions.

Segmented RNA viruses have a marked ability to affect molecular evolution through genome shuffling, which promotes genetic diversity and leads to distinct phylogenetic segregation of individual genome segments [44]. Genome exchange among hantaviruses predominantly occurs within and between closely related lineages [45,46]. Of particular interest is the observation that reassortment events frequently involve the M segment, suggesting that while the S and L segments require a higher degree of genetic compatibility, the M segment may either be more permissive for exchange or confer a selective advantage when reassorted [47]. For instance, SNV undergoes genetic reassortment in natural environments and *in vitro*, particularly affecting the M segment [48,49]. In the case of *Orthohantavirus andesense*, the substantial molecular diversity within the M segment resulted in five distinct clades, each corresponding to a different geographic region across South America [50]. Additionally, *in vitro* studies have documented reassortment of the DOBV M segment between distinct genotypes carried by different natural reservoir hosts [51]. However, reassortment involving the S or L segments has been observed in HTNV, SEOV, and PUUV, indicating that the degree of compatibility and tolerance for segment exchange may vary among different hantavirus species [10,52,53]. Herein, we identified three reassortant candidates (HTNV Aa22-84 and HTNV Aa22-95 from Paju-si, and HTNV Aa23-35 from Yeoncheon-gun) that exhibited a heterogeneous L segment, suggesting that the exchange of this segment may require lower compatibility thresholds and show greater tolerance than the S and M segments. To achieve a more comprehensive understanding of the dominance and evolutionary complexity of these reassortment events, future studies should prioritize continuous collection, sequencing, and analysis of diverse HTNV genome sequences across the ROK.

High-throughput sequencing is a robust tool for investigating genetic diversity within populations, and is crucial for understanding viral evolutionary dynamics and pathogenesis [32,54,55]. The reliability of consensus sequences depends on accurately accounting for the background errors inherent in sequencing technology and achieving the necessary sensitivity [56]. Sufficient sequencing coverage is essential for thoroughly characterizing viral populations [57]. Wang et al. showed that detecting minor variants within viral populations requires a minimum sequencing depth of 400× to identify variants at 1% frequency, and 1,000× for variants at 0.5% frequency, with 99.999% confidence [58]. Achieving these goals requires targeted amplification of viral genomes. Previous studies have indicated that nanopore sequencing using PCR-enriched libraries is an ideal, effective approach to obtain high-quality HTNV genomic sequences at the consensus level [27,37]. In this study, we developed a novel amplicon-based nanopore sequencing method using multiplex sampling runs to recover the complete genome sequence of HTNV, leading to high coverage rates and substantial sequencing depth. These improvements underscore the potential of the MinION platform to advance genomic surveillance and epidemiological research on hantaviruses, offering a more efficient and accessible method for generating comprehensive viral genome data.

Nanopore technology traditionally generates raw reads of lower quality than the Illumina platform, which has raised concerns regarding the reliability of sequence accuracy [59]. Previous studies have demonstrated that MinION sequencing using an R9 flow cell (ONT) produces unprocessed reads with high error rates, approximately 15% [60,61]. Although nanopore-based approaches have achieved substantial genome coverage and sequencing depth, they have been substantially hampered by mechanical indel errors, especially in homopolymer regions with earlier R9 chemistry [29–31]. These high error rates and indels in early nanopore systems pose challenges to the reliability of downstream virome analyses. Furthermore, single-nucleotide indels within coding regions can lead to frameshift mutations, complicating accurate genome interpretation. Our earlier work demonstrated, for the first time, that full-length genomes of HTNV could be obtained directly from animal samples using multiplex PCR-based nanopore sequencing [27]. Building on this, subsequent research confirmed the feasibility of completing the entire workflow—from sample preparation to phylogenetic analysis—within three hours per sample using amplicon-based nanopore Flongle sequencing [37]. While these studies highlighted the utility and rapid turnaround time of nanopore sequencing, they were hindered by high indel error rates, particularly in homopolymer regions, due to the limitations of the R9 chemistry used at the time. These errors required manual correction during downstream bioinformatics analysis, reducing the scalability and reliability of the approach. To address these limitations, a novel nanopore sequencing strategy was developed using the latest R10 chemistry in combination with the KU-ONT-HTNV-consensus module, which was optimized for error correction. This updated method was designed to produce high-fidelity genomic sequences of HTNV. The enhanced R10 chemistry, coupled with improved bioinformatics tools for error correction, significantly reduced the error rates associated with nanopore sequencing. This advancement markedly improved the accuracy and reliability of NGS data, enabling complete and precise genome sequencing of HTNV. It underscores the potential of this work to not only enhance our understanding and management of hantaviruses but also to contribute valuable tools and insights for addressing a diverse array of neglected viral pathogens, thereby strengthening global efforts in virome surveillance.

This study has several aspects that warrant further refinement in future research. 1) Molecular testing was restricted to lung tissues from seropositive rodents, potentially overlooking individuals in the early stages of infection who had not yet seroconverted. 2) Resource limitations confined testing to a single tissue type, thereby constraining insights into the systemic distribution of hantaviruses. 3) The current analysis primarily employs a phylogenetic framework, emphasizing spatial relationships and genetic diversity. To transition toward a more robust phylogeographic approach, future studies should incorporate evolutionary rates, molecular clock models, and temporal data, while broadening testing to include multiple tissues and both seronegative and seropositive individuals. These advancements would significantly deepen our understanding of hantavirus evolutionary and ecological dynamics.

In conclusion, 15 complete genomic sequences of HTNV were retrieved from rodents in the Gyeonggi and Gangwon Provinces, regions with high HFRS prevalence. These sequences significantly enhance the phylogenetic resolution of HTNV in the ROK. Genome exchange analysis identified three novel reassortant candidates involving a heterogeneous L segment, suggesting lower genetic compatibility requirements and greater tolerance than those of the S and M segments. A novel amplicon-based nanopore sequencing approach was developed using the latest R10 chemistry and KU-ONT-HTNV-consensus module, achieving high coverage and sequencing depth for whole-genome sequencing of HTNV. This report provides important insights into nanopore-based diagnostics, genomic surveillance, and evolutionary dynamics of orthohantaviruses, which are crucial for developing effective strategies against HFRS outbreaks in the ROK.

## Supporting information

S1 Fig A geographic map of trapping areas where small mammals were captured in the Republic of Korea, 2022–2023. There were 11 collection sites: Pocheon-si, Paju-si, Yeoncheon-gun, and Yangju-si in Gyeonggi Province; Chuncheon-si, Cheorwon-gun, Hongcheon-gun, Inje-gun, Hwacheon-gun, and Yanggu-gun in Gangwon Province; and Gwangju Metropolitan City. The initial map was generated using Quantum Geographical Information System 3.10 for Mac and further modified using Adobe Illustrator CC 2019. The base layer of the map was sourced from https://www.naturalearthdata.com/ and is freely available for use in any project without the need for permission.
(TIF)

S2 Fig Total number of small mammals captured in the Republic of Korea (ROK), 2022–2023. This figure shows the total numbers of rodents and shrews collected across the ROK from 2022 to 2023. The small mammals captured represented nine species: 498 *Apodemus agrarius*, four *A. peninsulae*, 42 *Crocidura lasiura*, seven *C. shantungensis*, one *Sorex mirabilis*, 17 *Myodes regulus*, five *Tscherskia triton*, four *Micromys minutus*, and one *Tamias sibiricus*. A histogram illustrating these data was created using GraphPad Prism 9 and subsequently modified using Adobe Illustrator CC 2019.
(TIF)

S3 Fig Phylogeny based on the mitochondrial DNA cytochrome *b* (*CYTB*) gene of *Apodemus agrarius* collected in the Republic of Korea, 2022–2023. This figure presents a phylogenetic tree constructed from the mitochondrial DNA *CYTB* gene sequences (positions 129–1,041 nt) of striped field mice (*A. agrarius*) captured during this study. The identification of *A. agrarius* was confirmed using conventional polymerase chain reaction targeting the mitochondrial DNA *CYTB* gene. Phylogenetic analysis was performed using the maximum likelihood method in MEGA 7.0. Branch lengths in the tree represent the number of nucleotide substitutions, with vertical distances adjusted for better visual clarity. The bootstrap probabilities calculated from 1,000 iterations, are indicated for each node. In this figure, the genomic sequences of the *CYTB* gene from *A. agrarius* are displayed, with the newly obtained sequences emphasized in bold red letters.
(TIF)

S4 Fig Genome coverage rates of amplicon-based nanopore sequencing for *Orthohantavirus hantanense* (HTNV) S, M, and L segments by viral copy number. The coverage rate was calculated by mapping the viral reads to the tripartite genomic sequences of HTNV 76-118 strain (GenBank accession numbers NC_005218, NC_005219, and NC_005222). S, small; M, medium; L, large.
(TIF)

S1 Table Accession number of genomic sequences of *Orthohantavirus hantanense* (HTNV) S, M, and L segments in this study. (PDF)

S2 Table Characteristics of *Orthohantavirus hantanense* (HTNV)-infected *Apodemus agrarius* collected in the Republic of Korea, 2022–2023. (PDF)

S3 Table Summary of mapped reads and average depth of multiplex polymerase chain reaction-based nanopore sequencing for *Orthohantavirus hantanense* (HTNV). (PDF)
