# Supplementary figures and images for: Epidemiological surveillance and phylogenetic diversity of Orthohantavirus hantanense using high-fidelity nanopore sequencing, Republic of Korea
Source: PLoS Negl Trop Dis. 2025 Feb 7;19(2):e0012859. doi: 10.1371/journal.pntd.0012859 (PMC11828426; doi:10.1371/journal.pntd.0012859)

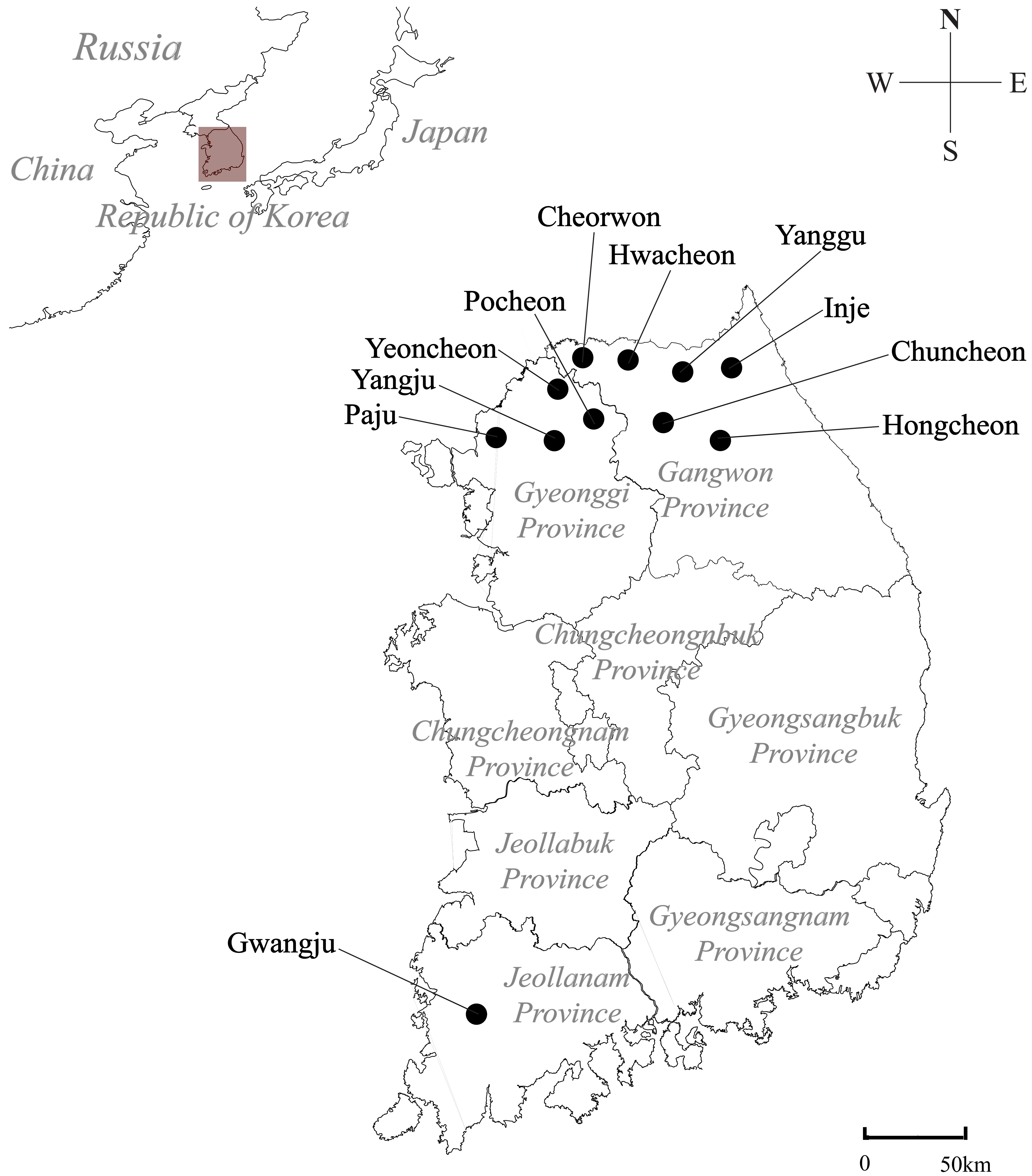

Supplement: S1 Fig — There were 11 collection sites: Pocheon-si, Paju-si, Yeoncheon-gun, and Yangju-si in Gyeonggi Province; Chuncheon-si, Cheorwon-gun, Hongcheon-gun, Inje-gun, Hwacheon-gun, and Yanggu-gun in Gangwon Province; and Gwangju Metropolitan City. The initial map was generated using Quantum Geographical Information System 3.10 for Mac and further modified using Adobe Illustrator CC 2019. The base layer of the map was sourced from https://www.naturalearthdata.com/ and is freely available for use in any project without the need for permission. (TIF) [file pntd.0012859.s001.tif]

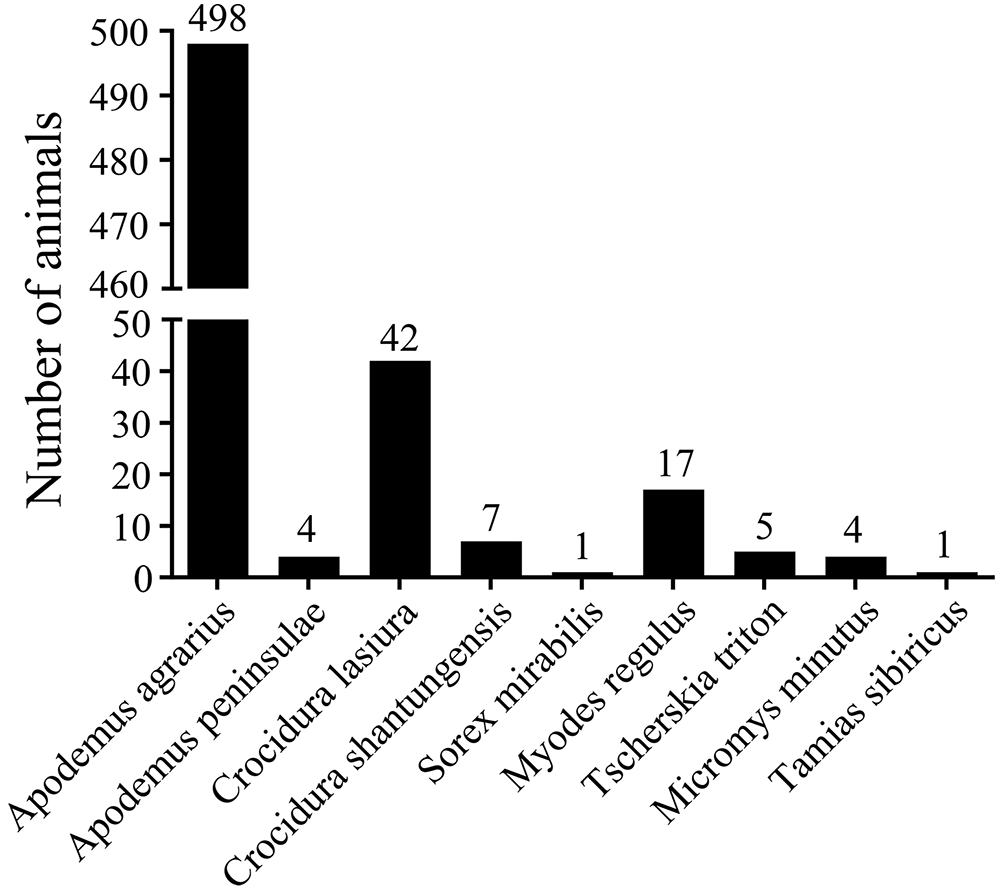

Supplement: S2 Fig — This figure shows the total numbers of rodents and shrews collected across the ROK from 2022 to 2023. The small mammals captured represented nine species: 498 Apodemus agrarius, four A. peninsulae, 42 Crocidura lasiura, seven C. shantungensis, one Sorex mirabilis, 17 Myodes regulus, five Tscherskia triton, four Micromys minutus, and one Tamias sibiricus. A histogram illustrating these data was created using GraphPad Prism 9 and subsequently modified using Adobe Illustrator CC 2019. (TIF) [file pntd.0012859.s002.tif]

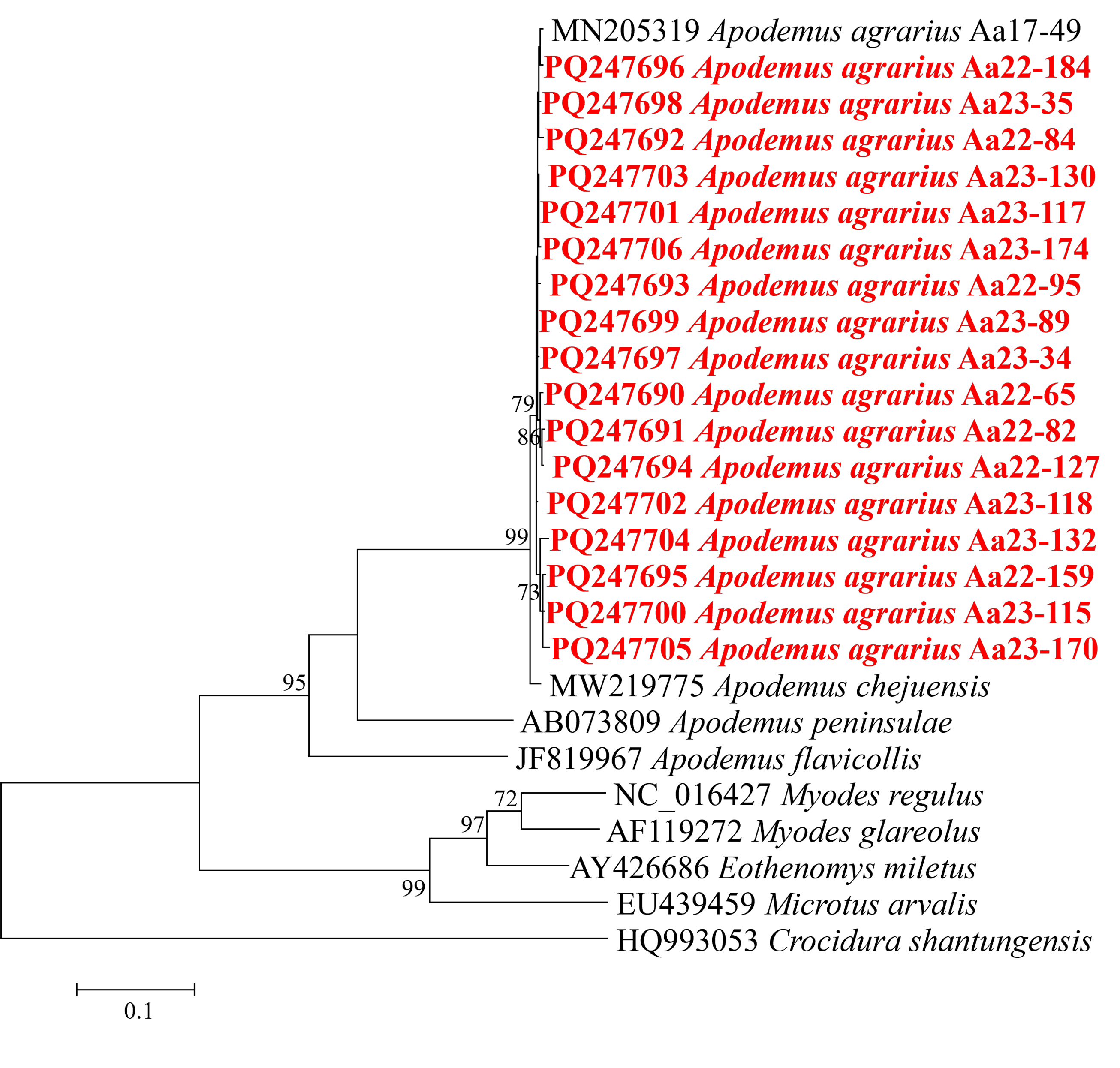

Supplement: S3 Fig — This figure presents a phylogenetic tree constructed from the mitochondrial DNA CYTB gene sequences (positions 129–1,041 nt) of striped field mice (A. agrarius) captured during this study. The identification of A. agrarius was confirmed using conventional polymerase chain reaction targeting the mitochondrial DNA CYTB gene. Phylogenetic analysis was performed using the maximum likelihood method in MEGA 7.0. Branch lengths in the tree represent the number of nucleotide substitutions, with vertical distances adjusted for better visual clarity. The bootstrap probabilities calculated from 1,000 iterations, are indicated for each node. In this figure, the genomic sequences of the CYTB gene from A. agrarius are displayed, with the newly obtained sequences emphasized in bold red letters. (TIF) [file pntd.0012859.s003.tif]

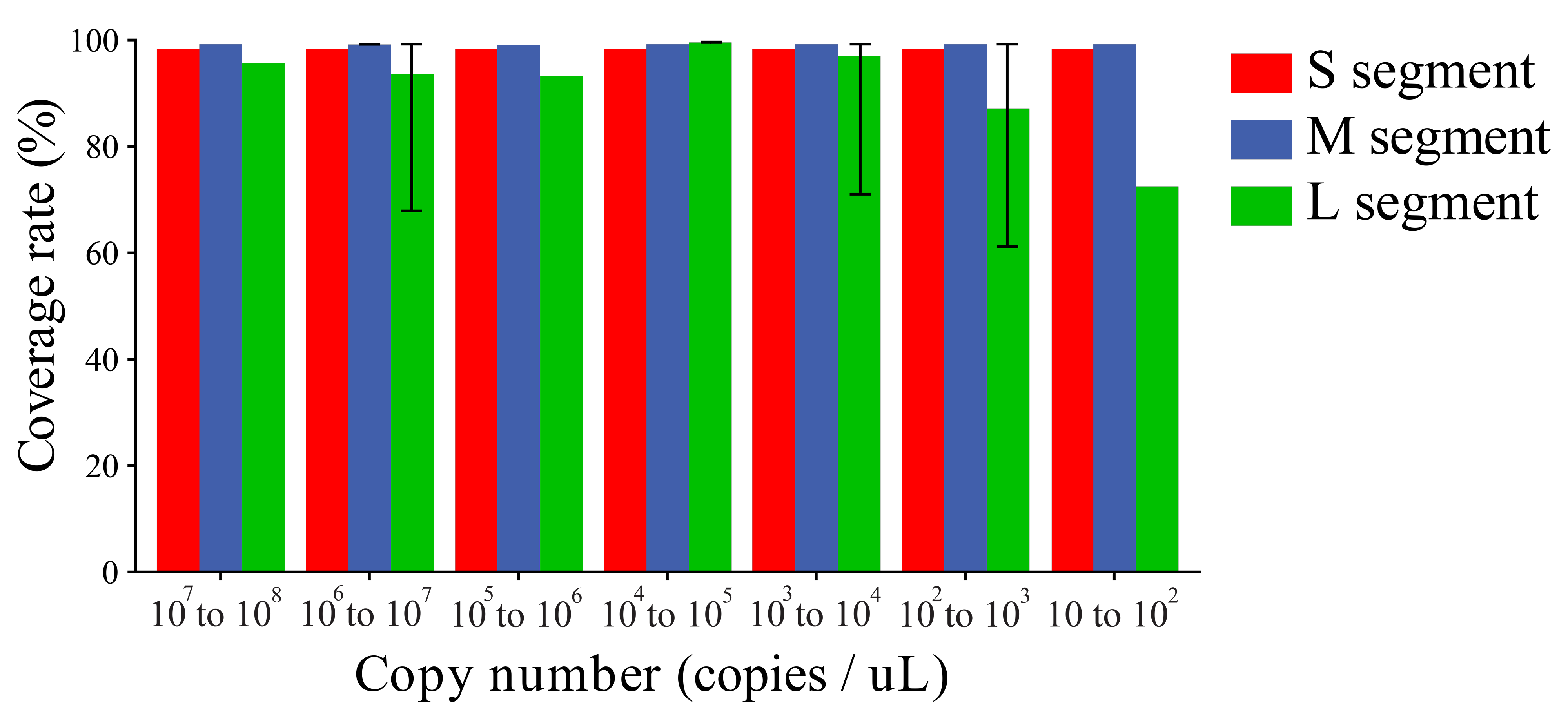

Supplement: S4 Fig — The coverage rate was calculated by mapping the viral reads to the tripartite genomic sequences of HTNV 76-118 strain (GenBank accession numbers NC_005218, NC_005219, and NC_005222). S, small; M, medium; L, large. (TIF) [file pntd.0012859.s004.tif]
