# Supplementary material for: Epidemiological surveillance and phylogenetic diversity of Orthohantavirus hantanense using high-fidelity nanopore sequencing, Republic of Korea
Source: PLoS Negl Trop Dis. 2025 Feb 7;19(2):e0012859. doi: 10.1371/journal.pntd.0012859 (PMC11828426; doi:10.1371/journal.pntd.0012859)
Supplement: S1 Table — (PDF) [file pntd.0012859.s005.pdf]

S1 Table. Accession number of genomic sequences of *Orthohantavirus hantanense* (HTNV) S, M, and L segments in this study

Strain	Year	Site	Nation	Accession No.		
				S segment	M segment	L segment
HTNV 76-118	1976	Dongducheon	ROK	M14626	NC005219	NC005222
HTNV Aa03-161	2003	Yeoncheon	ROK	KT935024	KT934990	KT934956
HTNV Aa03-386	2003	Yeoncheon	ROK	KT935025	KT934991	KT934957
HTNV Aa05-331	2005	Yeoncheon	ROK	KT935030	KT934996	KT934962
HTNV Aa10-123	2010	Paju	ROK	KT935036	KT935002	KT934968
HTNV Aa10-265	2010	Paju	ROK	KU207194	KU207186	KU207178
HTNV HV004	2012	Hubei	China	JQ083395	JQ083394	JQ083393
HTNV Aa14-184	2014	Paju	ROK	KU207171	KU207164	KU207157
HTNV Aa14-188	2014	Paju	ROK	KT935043	KT935009	KT934975
HTNV Aa15-69	2015	Hwacheon	ROK	MT012546	MT012558	MT012570
HTNV Aa15-84	2015	Hwacheon	ROK	MT012549	MT012561	MT012573
HTNV Aa17-49	2017	Yeoncheon	ROK	MH598506	MH598492	MH598478
HTNV Aa17-53	2017	Yeoncheon	ROK	MH598507	MH598493	MH598479
HTNV Aa19-36	2019	Cheorwon	ROK	MW796134	MW796122	MW796110
HTNV Aa19-38	2019	Cheorwon	ROK	MW796135	MW796123	MW796111
HTNV Aa19-89	2019	Yeoncheon	ROK	MW796138	MW796126	MW796114
HTNV Aa19-152	2019	Hwacheon	ROK	MW796140	MW796128	MW796116
HTNV Ac20-5	2020	Jeju	ROK	MW219769	MW219763	MW219757
HTNV Aa22-65	2022	Inje	ROK	PQ247675	PQ247660	PQ212937
HTNV Aa22-82	2022	Yeoncheon	ROK	PQ247676	PQ247661	PQ212938
HTNV Aa22-84	2022	Paju	ROK	PQ247677	PQ247662	PQ212939
HTNV Aa22-95	2022	Paju	ROK	PQ247678	PQ247663	PQ212940
HTNV Aa22-127	2022	Hwacheon	ROK	PQ247679	PQ247664	PQ212941
HTNV Aa22-159	2022	Cheorwon	ROK	PQ247680	PQ247665	PQ212942

HTNV Aa22-184	2022	Yeoncheon	ROK	PQ247681	PQ247666	PQ212943
HTNV Aa23-35	2023	Yeoncheon	ROK	PQ247682	PQ247667	PQ212944
HTNV Aa23-115	2023	Cheorwon	ROK	PQ247683	PQ247668	PQ212945
HTNV Aa23-117	2023	Cheorwon	ROK	PQ247684	PQ247669	PQ212946
HTNV Aa23-118	2023	Cheorwon	ROK	PQ247685	PQ247670	PQ212947
HTNV Aa23-130	2023	Cheorwon	ROK	PQ247686	PQ247671	PQ212948
HTNV Aa23-132	2023	Cheorwon	ROK	PQ247687	PQ247672	PQ212949
HTNV Aa23-170	2023	Paju	ROK	PQ247688	PQ247673	PQ212950
HTNV Aa23-174	2023	Paju	ROK	PQ247689	PQ247674	PQ212951

ROK, Republic of Korea; N.D, No data.
