# Supplementary material for: Epidemiological surveillance and phylogenetic diversity of Orthohantavirus hantanense using high-fidelity nanopore sequencing, Republic of Korea
Source: PLoS Negl Trop Dis. 2025 Feb 7;19(2):e0012859. doi: 10.1371/journal.pntd.0012859 (PMC11828426; doi:10.1371/journal.pntd.0012859)
Supplement: S2 Table — (PDF) [file pntd.0012859.s006.pdf]

PLOS NEGLECTED TROPICAL DISEASES

S2 Table. Characteristics of *Orthohantavirus hantanense* (HTNV)-infected *Apodemus agrarius* collected in the Republic of Korea, 2022–2023

Sample	Collection date	Collection site (City/Province)	Sex	Weight (g)	Anti-HTNV IgG titer	RT-PCR positivity	Vital status[a]
Aa22-65	Apr. 20, 2022	Inje-gun/Gangwon	M	21.5	1:2,048[b]	Pos	Dead
Aa22-82	Apr. 28, 2022	Yeoncheon-gun/Gyeonggi	F	22.5	1:4,096[b]	Pos	Alive
Aa22-84	May. 4, 2022	Paju-si/Gyeonggi	M	27.7	1:128[c]	Pos	Dead
Aa22-95	May. 4, 2022	Paju-si/Gyeonggi	M	31.0	1:1,024[b]	Pos	Alive
Aa22-127	May. 27, 2022	Hwacheon-gun/Gangwon	F	21.2	1:256[c]	Pos	Dead
Aa22-159	Jun. 16, 2022	Cheorwon-gun/Gangwon	M	28.8	1:512[c]	Pos	Dead
Aa22-184	Jul. 12, 2022	Yeoncheon-gun/Gyeonggi	M	44.5	1:512[b]	Pos	Alive
Aa23-34	Mar. 28, 2023	Yeoncheon-gun/Gyeonggi	F	34.2	1:256[b]	Pos	Alive
Aa23-35	Mar. 28, 2023	Yeoncheon-gun/Gyeonggi	M	36.1	1:512[b]	Pos	Alive
Aa23-89	Mar. 29, 2023	Yeoncheon-gun/Gyeonggi	F	26.7	1:256[c]	Pos	Dead
Aa23-115	Apr. 20, 2023	Cheorwon-gun/Gangwon	M	23.5	1:4[c]	Pos	Dead
Aa23-117	Apr. 20, 2023	Cheorwon-gun/Gangwon	M	33.3	1:128[c]	Pos	Dead
Aa23-118	Apr. 20, 2023	Cheorwon-gun/Gangwon	F	19.5	1:128[c]	Pos	Dead
Aa23-130	Apr. 20, 2023	Cheorwon-gun/Gangwon	M	21.4	1:256[c]	Pos	Dead
Aa23-132	Apr. 20, 2023	Cheorwon-gun/Gangwon	M	25.6	1:128[c]	Pos	Dead
Aa23-170	Mar. 17, 2023	Paju-si/Gyeonggi	F	23.8	1:32[c]	Pos	Dead
Aa23-174	Mar. 17, 2023	Paju-si/Gyeonggi	M	22.9	1:8[c]	Pos	Dead

[a], The vital status of the rodent, whether alive or dead at the time of capture, was determined; [b], Indirect immunofluorescence antibody (IFA) test was performed on sera; [c], The IFA test was performed on heart fluids. IgG, immunoglobulin G; RT-PCR, reverse transcription-polymerase chain reaction; Aa, *Apodemus agrarius*; M, male; F, female; Pos, positive.
