# Supplementary material for: Epidemiological surveillance and phylogenetic diversity of Orthohantavirus hantanense using high-fidelity nanopore sequencing, Republic of Korea
Source: PLoS Negl Trop Dis. 2025 Feb 7;19(2):e0012859. doi: 10.1371/journal.pntd.0012859 (PMC11828426; doi:10.1371/journal.pntd.0012859)
Supplement: S3 Table — (PDF) [file pntd.0012859.s007.pdf]

PLOS NEGLECTED TROPICAL DISEASES

S3 Table. Summary of mapped reads and average depth of multiplex polymerase chain reaction-based nanopore sequencing for *Orthohantavirus hantanense* (HTNV)

Viral RNA copy number (copies/uL)	Sample	Total reads	Reads mapped/ Total reads (%)	HTNV					
				S segment		M segment		L segment	
				Reads mapped	Depth of coverage[a]	Reads mapped	Depth of coverage[a]	Reads mapped	Depth of coverage[a]
10^7 to 10^8	Aa22-65	63,002	52,001 (82.5)	5,701	2,215.0	14,068	2,522.4	32,230	2,493.0
10^6 to 10^7	Aa23-132	91,715	85,366 (93.1)	19,763	9,997.9	27,827	7,082.7	37,738	6,529.2
	Aa22-84	43,811	39,609 (90.4)	4,289	2,168.9	14,937	3,107.8	20,383	2,071.6
	Aa23-34	23,602	823 (3.5)	143	63.6	271	44.9	409	27.0
	Aa23-130	122,317	116,350 (95.1)	28,851	14,125.5	41,584	9,192.2	45,900	6,576.2
	Aa22-95	56,414	46,606 (82.6)	3,614	1,586.5	22,235	3,169.5	20,756	1,909.5
	Aa23-35	43,128	24,928 (57.8)	1,753	557.0	11,667	727.0	11,508	461.0
	Aa23-117	174,764	156,639 (89.6)	62,315	28,263.0	52,477	7,118.7	41,726	4,172.9
10^5 to 10^6	Aa23-118	92,773	39,485 (42.6)	2,334	285.7	30,740	3,127.5	6,410	495.9
10^4 to 10^5	Aa22-159	70,047	44,926 (64.1)	7,212	3,715.0	6,527	1,032.1	31,182	3,422.8
	Aa22-82	33,298	26,440 (79.4)	2,388	1,209.8	8,793	1,691.9	15,258	1,605.7
10^3 to 10^4	Aa23-170	101,706	86,532 (85.1)	21,792	11,530.5	35,293	9,534.2	29,443	3,924.8
	Aa22-127	16,587	10,487 (63.2)	2,430	1,148.4	946	235.7	7,110	465.2
	Aa23-115	34,682	26,264 (75.7)	9,460	3,273.8	13,495	1,852.6	3,308	327.6
10^2 to 10^3	Aa23-174	52,681	48,272 (91.6)	15,768	8,482.3	21,270	5,782.2	11,231	1,414.0
	Aa22-184	55,575	39,151 (70.4)	2,503	1,354.0	12,327	2,906.9	24,321	2,752.5
10 to 10^2	Aa23-89	56,569	47,624 (84.2)	9,520	5,000.0	13,994	3,242.0	24,102	2,880.0
**Total**	**Average**	**66,628**	**52,441 (78.7)**	**11,755 (17.6%)**	**5,587**	**19,321 (29.0%)**	**3,669**	**21,354 (32.0)**	**2,443**

[a]: The depth of coverage was calculated as the number of mapped reads (read length × number of reads matching the reference/reference genome size). Aa, *Apodemus agrarius*.
